# Camphor as an Antidote to Strychnia

**Published:** 1855-02

**Authors:** 


*Camphor as an Antidote to Strychnia.*—Dr. S. H. Tewksbury, of Portland, Me., publishes in the Boston Medical and Surgical Journal, two cases of poisoning by strychnia. The first of these, a paraplegic patient, 22 years of age, of full habit, took by mistake about three-fourths of a grain of strychnia. Tetanic convulsions followed of great severity. During an interval of the paroxysms two teaspoonfulls of tinct. camph. were given. This was repeated at the next interval. In twenty minutes from the second dose the patient had only a sense of extreme fatigue remaining, which soon departed.

The second case was a boy of 12 years, who had eaten a biscuit intended for rats, containing about one and a half grains of strychnia. Owing to the lock-jaw present camphor could not be introduced into the stomach. It was accordingly injected, and the patient enveloped in a warm camphor bath. "The effect was happy and decided." In a few hours he was apparently well.

Dr. Tewksbury has also experimented upon dogs with this poison, and finds that camphor has an antidote power with them, when administered sufficiently freely and early. He was led to its use in the first case from the fact that it was the only antispasmodic at hand. This accidental use seems to have resulted in a full conviction of the efficacy of the remedy.

How far continued experience may support this new antidote for strychnia we cannot tell. The evidence in its favor is better than any other in our acquaintance, and it is certainly worthy of a trial.

Should it prove successful in this use it should then be freely resorted to in tetanus. The tetanic convulsion and that of strychnia are so similar, that the remedy for one would probably be appropriate for the other. The difference in causation, however, might interfere. The one is a poison in the blood which may soon be eliminated; the other is we know not what, a mysterious impression on the motor nerves, whose mode of action is not yet reached. The horrors of tetanus are such as to warmly interest the physician in a search for its cure. We have several times witnessed deaths in tetanic convulsion. In one, the last we have seen, death occurred instantaneously at the beginning of a paroxysm. During the interval preceding it the unfortunate patient had spoken calmly and cheerfully. He was interrupted by the spasm; his jaw first, then the muscles of the thorax, then the diaphragm became fixed, his chest was like iron, and he died as suddenly as if choked. Asphyxia was the immediate cause of death. H.

